# Shared Genetic Risk Factors Between Cancer and Cardiovascular Diseases

**DOI:** 10.3389/fcvm.2022.931917

**Published:** 2022-07-07

**Authors:** Aleksander Turk, Tanja Kunej

**Affiliations:** Department of Animal Science, Biotechnical Faculty, University of Ljubljana, Domžale, Slovenia

**Keywords:** cancer, cardiovascular disease, risk factor, chemotherapy, comorbidity, genetic variant, interaction network, cardio-oncology

## Abstract

Cancer and cardiovascular diseases (CVD) account for approximately 27.5 million deaths every year. While they share some common environmental risk factors, their shared genetic risk factors are not yet fully understood. The aim of the present study was to aggregate genetic risk factors associated with the comorbidity of cancer and CVDs. For this purpose, we: (1) created a catalog of genes associated with cancer and CVDs, (2) visualized retrieved data as a gene-disease network, and (3) performed a pathway enrichment analysis. We performed screening of PubMed database for literature reporting genetic risk factors in patients with both cancer and CVD. The gene-disease network was visualized using Cytoscape and the enrichment analysis was conducted using Enrichr software. We manually reviewed the 181 articles fitting the search criteria and included 13 articles in the study. Data visualization revealed a highly interconnected network containing a single subnetwork with 56 nodes and 146 edges. Genes in the network with the highest number of disease interactions were *JAK2*, *TTN, TET2*, and *ATM*. The pathway enrichment analysis revealed that genes included in the study were significantly enriched in DNA damage repair (DDR) pathways, such as homologous recombination. The role of DDR mechanisms in the development of CVDs has been studied in previously published research; however, additional functional studies are required to elucidate their contribution to the pathophysiology to CVDs.

## Introduction

Cancer and cardiovascular diseases (CVD) are among the leading causes of death worldwide. An estimated 17.9 million people die every year as a result of CVDs and approximately 9.6 million deaths per year are caused by cancer (1, 2). While they are two distinct types of illness, they display a significant level of comorbidity (3). A portion of this comorbidity has been attributed to the cardiotoxic effects of chemotherapeutic cancer treatments (4). Cancer and CVD also share contributing risk factors, such as hypertension, obesity, smoking, diabetes mellitus and lifestyle choices, as well as pathophysiological mechanisms, such as oxidative stress, neuro-hormonal activation and inflammation (5). The identification of these common risk factors and mechanisms has led to increased focus on the various possible connections between the two diseases in the field of cardio-oncology. However, much of this research has been focused on the effects of medication and common risk factors. As a result, potential underlying genetic contributors to this comorbidity are poorly understood.

Among the known genetic factors that contribute to the development of both cancer and CVDs is clonal hematopoiesis of intermediate potential (CHIP). CHIP refers to the presence of a sub-population of clonally expanded hematopoietic stem cells within an individual. This may occur as a result of age-related genetic drift or due to genetic variants. While the presence of CHIP is not inherently malignant, it does confer a higher risk for the development of blood malignancies, such as leukemia and CVDs. CHIP has also been associated with worsened heart failure outcomes (6). It has also been shown that patients with congenital heart disease (CHD) carry more damaging gene variants in cancer risk genes, suggesting shared biological pathways (7). Associations between cancer and CVDs on a genetic level have therefore already been identified. Further exploration into their shared genetic background could thus yield valuable results and contribute to our understanding of both cancer and CVDs.

The aim of the present study was to aggregate genetic risk factors associated with the comorbidity of cancer and CVDs. Our goal is to visualize gene-disease interactions in genes with variants associated with cancer and CVD comorbidity. Additionally, the goal is to perform a protein-protein interaction (PPI) analysis for genes included in the study and to perform pathway enrichment analysis to identify biological pathways in which disease-associated genes are involved. This would contribute to the field of cardio-oncology by compiling genetic risk factors and their roles for multiple disease types, which could be used for future development of risk assessment, more accurate prognoses and personalized treatment choices.

## Materials and Methods

The PubMed database was accessed in order to collect relevant articles. Four database searches were conducted, containing common keywords pertinent to the subject matter:

1. Cardiac, mutations, risk, prevalence, predispose*, cancer
2. Heart, cancer, mutation, risk, cardiooncology
3. Cardiovascular, propensity, mutation, risk factor, cancer
4. Heart, cancer, gene*, risk, cardiooncology

The articles within the search results were then manually reviewed and screened for studies that fit two criteria:

1. Both cancer and CVD phenotypes were observed in patients
2. The study is concerned with shared genetic risk factors for cancer and CVDs

Studies that fit both screening criteria were then included in the study. Cancer type, CVD type, chemotherapeutic methods, as well as names of genes with disease-associated variants, were retrieved from studies. STRING software was then used to identify protein-protein interactions (PPIs) between proteins encoded by genes included in the present study (8). Only PPIs categorized as “retrieved from curated databases” or “experimentally validated” were included. The PPIs, genes, and their associated cancer and cardiovascular conditions were then visualized as a network using Cytoscape software (9). Secondary literature review was then conducted for genes with the highest number of disease-associated interactions in the network.

**FIGURE 1:**
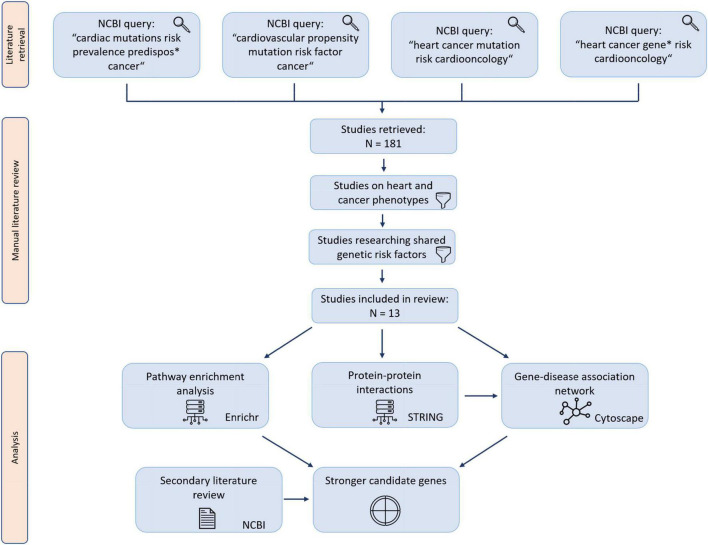
Study workflow diagram. Retrieved studies were manually reviewed for studies matching the screening criteria.

A pathway enrichment analysis using Enrichr was performed on genes included in this study, drawing from the BioPlanet 2019, KEGG 2021 and WikiPathway 2021 databases (10). In order to include the most reliable enrichments, pathway enrichment analysis results were then sorted by lowest adjusted *P*-value. The 10 lowest *P*-value results of each database were included in the study. A workflow diagram of the study is presented in Figure 1.

## Results

In this study, we conducted manual literature review of genes with variants associated with cancer and CVD comorbidity and then visualized this data as a gene-disease network. Additionally, we expanded upon the network with PPIs obtained from the STRING database. An analysis revealed the obtained gene list was significantly enriched in pathways responsible for DDR. Finally, we conducted secondary literature review of genes identified with the study methodology.

**FIGURE 2:**
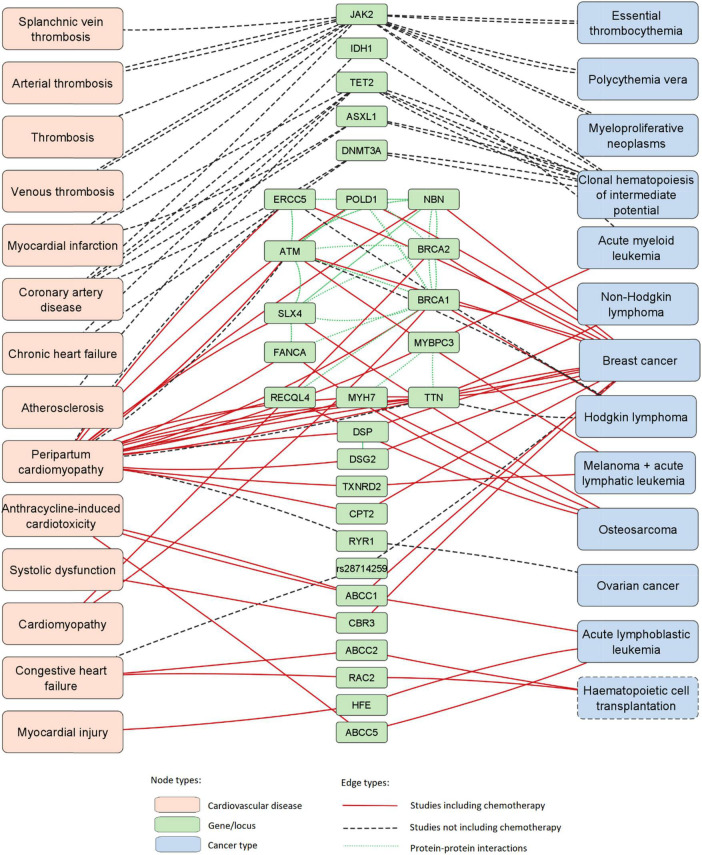
Network of associations between 29 loci (28 genes and 1 SNP), cancer types and CVDs. This network was expanded with data of PPIs. Hematopoietic cell transplantation is marked with a dashed node outline as it is not a disease.

The PubMed database was accessed with four queries containing common keywords for the field of cardio-oncology; the four queries combined yielded 181 articles as search results. After screening and manual review, 13 articles were included in the final data set (11–23). Out of 13 articles included in the study, 10 included chemotherapy as a study parameter. Data extracted from manually reviewed articles is available in Supplementary Table 1. A list of PPIs identified with STRING is available in Supplementary Table 2. Data visualization revealed a large interconnected network of associations between genes, cancer types and CVDs as well as PPIs (Figure 2). The network was composed of 29 loci (28 genes and 1 intergenic region), 13 cancer types and 14 CVDs; thus containing 56 nodes and 146 edges.

Genes in the network with the most disease-connected edges were *JAK2*, *TTN, TET2*, and *ATM*, with 16, 12, 8, and 6 edges, respectively. Diseases with the highest number of gene-connected edges were peripartum cardiomyopathy, breast cancer, clonal hematopoiesis of intermediate potential (CHIP) and coronary artery disease, with 24, 15, 10, and 5 edges, respectively.

Furthermore, we conducted a pathway enrichment analysis of the 28 disease-associated genes. Of the 30 pathway enrichments, 28 enrichments had an adjusted *P*-value ≤ 0.05 (Table 1 and Supplementary Table 3). Among them, 3 were associated with CVDs, 4 were associated with cancer, and 16 were associated with DNA damage repair. Other enrichments include nucleotide methylation, meiosis or meiotic recombination, membrane transport proteins and other functions.

**TABLE 1:** Pathway enrichment analysis for 28 genes associated with cancer and CVDs.

Pathway source	Pathway name	*P*-value	Adjusted *P*-value	Odds ratio	Combined score	Network genes included in pathway
Bioplanet 2019	BRCA1, BRCA2 and ATR roles in cancer susceptibility	1.15E-10	1.33E-08	244.53	5595.75	*FANCA, ATM, BRCA1, NBN, BRCA2*
Bioplanet 2019	DNA repair	1.27E-10	1.33E-08	64.52	1470.09	*POLD1, FANCA, ATM, ERCC5, BRCA1, NBN, BRCA2*
Bioplanet 2019	ATM-mediated phosphorylation of repair proteins	2.74E-08	0.000001138	1152.06	20062.49	*ATM, NBN, BRCA2*
Bioplanet 2019	Double-strand break repair	2.56E-08	0.000001138	177.36	3100.38	*ATM, BRCA1, NBN, BRCA2*
Bioplanet 2019	Fanconi anemia pathway	2.56E-08	0.000001138	177.36	3100.38	*FANCA, ATM, BRCA1, BRCA2*
Bioplanet 2019	Homologous recombination	8.25E-08	0.000002453	127.65	2082.04	*POLD1, ATM, NBN, BRCA2*
Bioplanet 2019	BARD1 signaling events	8.25E-08	0.000002453	127.65	2082.04	*POLD1, ATM, NBN, BRCA1*
Bioplanet 2019	Recruitment of repair and signaling proteins to double-strand breaks	1.53E-07	0.000003971	460.75	7231.36	*ATM, BRCA1, NBN*
Bioplanet 2019	Meiotic recombination	0.000001072	0.00002477	63.75	876.27	*NBN, ATM, BRCA1, BRCA2*
Bioplanet 2019	Meiosis	0.000006051	0.0001259	40.29	484.07	*NBN, ATM, BRCA1, BRCA2*
WikiPathways 2021	DNA Repair Pathways Full Network WP4946	3.28E-10	3.97E-08	55.92	1221.07	*POLD1, FANCA, ATM, ERCC5, BRCA1, NBN, BRCA2*
WikiPathways 2021	Homologous recombination WP186	2.53E-09	1.53E-07	354.88	7025.6	*POLD1, ATM, NBN, BRCA2*
WikiPathways 2021	DNA IR-damage and cellular response via ATR WP4016	9.94E-08	0.000004007	55.27	891.15	*FANCA, ATM, BRCA1, NBN, BRCA2*
WikiPathways 2021	DNA IR-double strand breaks and cellular response via ATM WP3959	0.000001155	0.00003493	62.49	854.39	*ATM, BRCA1, NBN, BRCA2*
WikiPathways 2021	ATM Signaling Pathway WP2516	0.00002612	0.0006321	62.16	656.01	*ATM, BRCA1, NBN*
WikiPathways 2021	DDX1 as a regulatory component of the Drosha microprocessor WP2942	0.00004244	0.0008558	295.79	2977.88	*ATM, NBN*
WikiPathways 2021	Cytosine methylation WP3585	0.00007262	0.001098	211.26	2013.35	*IDH1, TET2*
WikiPathways 2021	Breast cancer pathway WP4262	0.00006911	0.001098	21.14	202.54	*ATM, BRCA1, NBN, BRCA2*
WikiPathways 2021	Irinotecan pathway WP229	0.0001568	0.001725	134.41	1177.53	*ABCC1, ABCC2*
WikiPathways 2021	DNA damage response WP707	0.0001289	0.001725	35.34	316.48	*ATM, BRCA1, NBN*
KEGG 2021	Homologous recombination	3.22E-09	3.16E-07	115.36	2255.82	*POLD1, ATM, BRCA1, NBN, BRCA2*
KEGG 2021	Fanconi anemia pathway	0.000001072	0.00005252	63.75	876.27	*SLX4, FANCA, BRCA1, BRCA2*
KEGG 2021	ABC transporters	0.00003733	0.001219	54.75	558.21	*ABCC1, ABCC2, ABCC5*
KEGG 2021	Hypertrophic cardiomyopathy	0.0002958	0.007011	26.37	214.29	*MYBPC3, TTN, MYH7*
KEGG 2021	Dilated cardiomyopathy	0.0003577	0.007011	24.66	195.72	*MYBPC3, TTN, MYH7*
KEGG 2021	MicroRNAs in cancer	0.0009902	0.01617	10.28	71.13	*ABCC1, DNMT3A, ATM, BRCA1*
KEGG 2021	Nucleotide excision repair	0.002107	0.0295	32.8	202.12	*POLD1, ERCC5*
KEGG 2021	Viral myocarditis	0.003411	0.04178	25.43	144.47	*RAC2, MYH7*

## Discussion

In this study we conducted a database search and literature review for genes associated with cancer and CVD comorbidity, including chemotherapy-induced cardiotoxicity. Of the 181 manually reviewed articles, 13 were included in the analysis. We then formed a network of gene-disease associations, which was expanded upon with PPI data. Analysis revealed a large interconnected network containing a single subnetwork with 56 nodes and 146 edges. Enrichment analysis results also showed that genes were enriched in pathways associated with DNA damage repair, cancer and CVDs. Genes in the gene-disease association network with the highest number of edges were *JAK2*, *TTN, TET2*, and *ATM.*

Janus kinase 2 (JAK2) is encoded by the *JAK2* gene. It serves as a kinase for cytokine receptors and is part of the JAK-STAT signaling pathway, which allows the cell to respond to various stress factors, including endotoxins, hypoxia, UV radiation and hyperosmolarity (24). The pathway is also important for the regulation of the immune system through the polarization of T helper cells (25). JAK2 plays an important role in hematopoiesis, as *JAK2* gene disruptions in animal models are embryonically lethal due to a lack of definitive erythropoiesis (26). *JAK2* has been associated with the copresence of CHIP and myocardial infarction (16). A variant of *JAK2* has also been associated with an increased risk of developing myeloproliferative neoplasms such as polycythemia vera, essential thrombocythemia and primitive myelofibrosis (18, 27). As such JAK2 has been proposed as a potential target for the treatment or management of these diseases (28).

The *TTN* gene encodes titin (TTN), a large protein that plays a structural, scaffolding and signaling role in sarcomeres of striated muscle tissue (29). TTN spans from the Z-disc to the M-band, functioning as a molecular spring and giving muscles passive stiffness (30). The protein is also present in the heart’s muscle tissue. While its mechanical and scaffolding properties are critical for sarcomere function, its signaling role is also important. TTN binds to telethonin via its NH_2_ terminal domain, thus recruiting the muscle LIM protein (MLP) to the Z-line, where it interacts with other signaling molecules (31, 32). TTN could also be involved in hypertrophic signaling through its N2-A domain. This domain interacts with multiple signaling molecules, including muscle-ankyrin-repeat-proteins (MARPs), which are stress-response molecules and signal transducers (31). *TTN* variants have been associated with several CVDs, including dilated cardiomyopathy, fatal cardiomyopathy and heart failure (29, 33, 34). *TTN* has also been associated with peripartum cardiomyopathy, Hodgkin lymphoma, non-Hodgkin lymphoma and breast cancer (12). Furthermore, it has been suggested that the mutation load of *TTN* is indicative of a high tumor mutation burden (35).

Tet methylcytosine dioxygenase 2 (TET2), encoded by *TET2*, plays a role in epigenetic DNA modification by converting 5-methylcytosine to 5-hydroxymethylcytosine, thus promoting DNA demethylation (36). Through its regulatory functions TET2 affects hematopoiesis by promoting the self-renewal of stem cells, lineage commitment and monocyte differentiation (37). Li et al. report that 8% of *TET2*-knockout mice developed lethal myeloid malignancies within their first year of life (38). *TET2* variants have also been associated with increased risk of myeloid malignancies. These malignancies include B- and T-cell lymphomas, myeloproliferative neoplasms, chronic myelomonocytic leukemia, acute myeloid leukemia, myelodysplastic syndrome, CHIP and others (16, 19, 36, 38). Sano et al. have shown that *TET2* deficiency in mice is associated with greater cardiac dysfunction and heart failure, accelerated by *TET2-*induced clonal hematopoiesis. This is likely caused by the influence of *TET2* deficiency on the IL-1β/NLRP3 inflammatory pathway, as treatment with an NLRP3 inflammation inhibitor protected against the development of heart failure (39).

The *ATM* gene encodes the ATM serine/threonine kinase (ATM), which regulates multiple processes, including DNA damage response, oxidative stress levels and mitochondrial homeostasis (40). ATM plays a role in the cellular response to double-stranded breaks (DSB) (41). It phosphorylates TP53 – one of the most important tumor suppressor proteins (41, 42). Furthermore, ATM has also been shown to phosphorylate BRCA1, NBN and other tumor suppressor proteins (43, 44). *ATM* variants have been associated with a predisposition for developing ischemic heart disease (45). Beside CVDs, *ATM* variants have been associated with conveying an increased risk of developing some cancers, including chronic lymphocytic leukemia, breast cancer, pancreatic cancer and mantle cell lymphoma (12, 46, 47). While ATM has several tumor suppressing functions, ATM*-*induced signaling can cause tumor progression via the αvβ3 integrin pathway in some cancers (48). ATM-dependent signaling in tumor cells can also increase resistance to radiotherapy and chemoresistance (49). Due to its role in signaling pathways and DNA damage repair, ATM has been proposed as a potential target for the development of future chemotherapies (50).

In the present study, a pathway analysis revealed that the genes included in the analysis were enriched in pathways associated with DDR. Among them were pathways such as Nucleotide excision repair, DNA damage response WP707 and Double-strand break repair. While variants of DDR genes are known to contribute to cancer development, they have also been associated with some CVDs, such as heart failure and atherosclerosis (51). Results of a study of DDR in experimental heart failure in rats conducted by Yndestad et al. suggest that DDR mechanisms could play an important part in counteracting CVD-related genotoxic stress and damage to tissue (52). DDR mechanisms are important for counteracting the effects of oxidative stress on the cell (51). Among other shared risk factors, increased oxidative stress contributes to the development of both cancer and CVDs (53). Variants of DDR genes could thus be related to an increased susceptibility to damage from endogenous oxidative stress. Additional functional research is necessary to determine the role of DDR mechanisms in CVDs.

Several genes included in this study were identified in research concerned with the effects of chemotherapies on CVD development. Chemotherapeutics are a diverse group of molecules that remains a widely used approach in cancer treatment. These molecules often target rapidly replicating cancer cells by inducing DNA damage (54). However, this treatment also affects other cells, leading to various side effects, including cardiotoxicity. A common class of chemotherapeutics are anthracyclines and are known to have dose-dependent cardiotoxic side effects (54). The main mechanism of anthracycline-induced cardiotoxicity (ACT) is thought to affect heart cells by topoisomerase 2β inhibition, leading to cardiomyocyte apoptosis and mitochondrial biogenesis inhibition (55). The mechanisms of cardiotoxicity are not equally understood in all chemotherapeutics, however. The pathophysiology of HER-2-targeting agents, used for the treatment of breast cancer, is currently unclear (56). Further studies into the mechanisms of chemotherapy-induced cardiotoxicity for various treatments could serve to improve future therapeutic developments.

Genes included in this study could serve as potential variant screening targets for chemotherapy-induced cardiotoxicity risk assessment. Aminkeng et al. have identified variants of several genes associated with ACT, which could also serve as potential screening targets (57). Gene panels may be more cost effective than whole genome sequencing (WGS) for this purpose, but lack the option of re-analysis as knowledge of genetic risk factors advances (58). Pharmacogenomic testing is recommended for childhood cancer patients with indication for the anthracyclines daunorubicin and doxorubicin for specific variants of *RARG*, *SLC28A3* and UGT1A6*4. However, testing is not currently recommended for adult patients or children with indications for other anthracycline treatment (57). As the cost of WGS and gene panels fall and risk assessment strategies improve, screening a larger number of patients may become viable.

Accurately predicting cardiotoxic effects in patients and elucidating their pathophysiological mechanisms are of vital importance in cardio-oncology. This requires processing large data quantities, which is a task well-suited for machine learning. In cardio-oncology studies, machine learning has been used to perform retrospective cardiotoxicity risk assessment (59) and to estimate the safety of hERG blockers (60). Computational approaches could also have potential to improve cardiovascular imaging (61). Current research also suggests that these methods could be useful for predicting the cardiotoxic effects of drugs (62, 63). The use of machine learning may, in the future, assist clinicians in identifying probable pathogenic pathways and the treatment option with the highest likelihood of success.

## Conclusion

In this study we conducted manual literature review and data synthesis of genetic factors associated with cancer and CVD comorbidity. We visualized a network of gene-disease associations based on data extracted from 13 articles. Additionally, we added information on PPIs of proteins encoded by genes in this study and included the PPIs in the network. The results revealed a large, highly interconnected network of known genetic risk factors shared between cancer and CVDs. Pathway enrichment analysis indicate that the genes included in this study are enriched in various DDR pathways, such as homologous recombination. While prior literature has associated some DDR gene variants with cancer and CVD comorbidity, the mechanism behind this has not yet been sufficiently studied. Additional functional studies are required to elucidate the role of DDR gene variants in the development of both cancer and CVDs and chemotherapy-induced cardiotoxicity. In the future, gene variants screening before cancer treatment could improve outcomes for patients at risk of cardiotoxicity.

## Study Limitations

Patient chemotherapy information was extracted from articles during manual literature review. Some articles analyzed in the present study did not include chemotherapeutics as a study parameter, as it was not the intended focus of the studies. While treatment was not a study parameter, patients may have undergone chemotherapeutic treatment, which could contribute to CVD development. Therefore, it is possible that chemotherapies contributed to disease development in these studies as well. Moreover, in this study we focused on changes at the DNA level. In the future, taking into account other omics levels, such as transcriptomics, proteomics and epigenomics may yield a more holistic view of the study field. It is also possible that some relevant research articles may not have been included based on the present study’s queries. Searches for specific associations between CVD and cancer types may yield more publications as a database search output. We highlighted a few genes with the highest number of edges in the gene-disease association network. It should be noted, however, that an increased number of connections may be due to research interest and it is possible that other genes in the network could also be of great significance.

## Future Developments

Variants of genes involved in DDR pathways appear to contribute to cancer and CVD comorbidity in cancer patients undergoing chemotherapy. Further functional studies would contribute to the understanding of the underlying disease mechanisms and allow for the development of precision medicine approaches to disease treatment.

The present study could serve as a basis for the development of a cardio-oncology database. Such a database could prove invaluable for the understanding of the molecular relationship between diseases and identifying potential novel molecular targets for treatment. Additionally, it could serve to identify biomarkers, which could be used for risk assessment and optimal treatment choices.

While cardio-oncology is a rapidly expanding field, many genetic risk factors shared by cancers and CVDs are currently not yet known. Thus, the network formed in this study is incomplete and will require follow-up studies as more research in the field is conducted.

## Data Availability Statement

The original contributions presented in this study are included in the article/Supplementary Material, further inquiries can be directed to the corresponding author.

## Ethics Statement

Ethical review and approval was not required for the study on human participants in accordance with the local legislation and institutional requirements. Written informed consent from the patients/participants or patients/participants legal guardian/next of kin was not required to participate in this study in accordance with the national legislation and the institutional requirements.

## Author Contributions

AT conducted literature review, screening and analysis, wrote the manuscript, and created graphics. AT and TK designed study methodology and conducted manuscript review. Both authors contributed to the article and approved the submitted version.

## Conflict of Interest

The authors declare that the research was conducted in the absence of any commercial or financial relationships that could be construed as a potential conflict of interest.

## Publisher’s Note

All claims expressed in this article are solely those of the authors and do not necessarily represent those of their affiliated organizations, or those of the publisher, the editors and the reviewers. Any product that may be evaluated in this article, or claim that may be made by its manufacturer, is not guaranteed or endorsed by the publisher.
